# Research Trends on Metabolic Syndrome in Digital Health Care Using Topic Modeling: Systematic Search of Abstracts

**DOI:** 10.2196/53873

**Published:** 2024-12-12

**Authors:** Kiseong Lee, Yoongi Chung, Ji-Su Kim

**Affiliations:** 1 Humanities Research Institute Chung-Ang University Seoul Republic of Korea; 2 Department of Nursing Chung-Ang University Seoul Republic of Korea

**Keywords:** metabolic syndrome, digital health care, topic modeling, text network analysis, research trends, topic modeling, prevention, management, telemedicine, wearable, devices, apps, applications, methodological, cardiovascular disease

## Abstract

**Background:**

Metabolic syndrome (MetS) is a prevalent health condition that affects 20%-40% of the global population. Lifestyle modification is essential for the prevention and management of MetS. Digital health care, which incorporates technologies like wearable devices, mobile apps, and telemedicine, is increasingly becoming integral to health care systems. By analyzing existing research trends in the application of digital health care for MetS management, this study identifies gaps in current knowledge and suggests avenues for future research.

**Objective:**

This study aimed to identify core keywords, topics, and research trends concerning the use of digital health care in the management of MetS.

**Methods:**

A systematic search of abstracts from peer-reviewed papers was conducted across 6 academic databases. Following eligibility screening, 162 abstracts were selected for further analysis. The methodological approach included text preprocessing, text network analysis, and topic modeling using the BERTopic algorithm.

**Results:**

Analysis of the 162 selected abstracts yielded a keyword network comprising 1047 nodes and 34,377 edges. The top 5 core keywords were identified as “MetS,” “use,” “patient,” “health,” and “intervention.” We identified 12 unique topics, with topic 1 focusing on the use of telehealth for self-management of diabetes. The diversity of the 12 topics reflected various aspects of digital health care, including telehealth for diabetes management, electronic health records for MetS complications, and wearable devices for monitoring metabolic status. Research trends showed an expanding field of precision medicine driven by the demand for tailored interventions and the significant impact of the COVID-19 pandemic.

**Conclusions:**

By analyzing past research trends and extracting data from scholarly databases, this study has provided valuable insights that can guide future investigations in the field of digital health care and MetS management.

## Introduction

Metabolic syndrome (MetS) is reported to have a global prevalence ranging between 20% and 40% [1]. Characterized as a cluster of major interrelated risk factors for cardiovascular disease, MetS significantly exacerbates health outcomes by more than doubling the risk of cardiovascular diseases [2]. A MetS diagnosis is established when 3 or more of the following 5 risk factors are present: waist circumference indicative of obesity, low high-density lipoprotein cholesterol, hypertriglyceridemia, hypertension, and hyperglycemia [3]. As MetS is closely linked to lifestyle factors such as poor diet and insufficient physical activity (PA) [4], it can be prevented by targeted lifestyle modifications [5]. Given the substantial individual, societal, and economic burdens MetS imposes, proactive prevention and ongoing management are imperative.

Digital health care refers to the use of information and communication technology to monitor and manage health and well-being [6]. Advances in the information and communication technology sector have facilitated health care delivery through mobile apps, wearable devices, artificial intelligence, the Internet of Things, and telemedicine [7]. Digital health care has already been extensively applied across various health care domains. For instance, smart bands, a category of wearable devices, can quantify PA metrics such as step count, walking distance, and walking intensity [8]. Studies indicate that the adoption of these wearable devices can enhance PA levels among individuals with chronic conditions [9]. Moreover, health-related mobile apps have the potential to instigate behavioral changes in users by providing educational content, personalized health counseling, and diet and exercise monitoring [10]. This approach takes genetic, environmental, and lifestyle factors into account to develop personalized treatment plans, offering potential improvements in the management of MetS through more targeted interventions [11]. In other words, it enables the application of precision medicine, a health care approach that tailors medical treatment to each patient’s individual characteristics [11]. Given that digital health care is already widely used in health care settings, evidence from multiple studies [1,5,7-10] supports its applicability in managing MetS-associated risk factors, including obesity and diabetes.

To mitigate the impact of MetS and enhance long-term quality of life through ongoing management, a deep understanding of existing research trends is crucial. Previous studies on MetS within the realm of digital health care have used systematic reviews, meta-analyses [9,12-14], and scoping reviews [15]. However, these approaches have limitations, particularly in the depth to which they explore subjects, key concepts, and topics. Given the rapid advancements in digital health care research, it is imperative that researchers stay informed about the evolving knowledge structure, including emerging topics and shifting trends in MetS and digital health care research. This understanding is essential for maintaining the relevance and efficacy of ongoing and future studies in this dynamic field.

This study used text network analysis to analyze the frequency and patterns of the co-occurrence of words in textual data such as article abstracts [16,17]. This method offers the benefit of allowing a visual and intuitive understanding of the relationships between significant keywords, serving as a valuable network statistical indicator [16,17]. Furthermore, topic modeling serves as a statistical inference model that uses text mining to objectively and clearly evaluate research topics [18]. We used text mining analysis to examine trends in MetS-related digital health care research to understand the existing knowledge structure and inform the direction of future investigations. Accordingly, this study aimed to clarify the relationships between keywords by analyzing previous research on MetS within the domain of digital health care through text network analysis and to investigate research topics related to MetS within the digital health care sector.

The objective of this study was to construct a network that consolidates prior research on MetS within the field of digital health care. The study aimed to create an informed body of literature by comparing and analyzing keywords and research topics. The study had 3 primary goals: first, to identify the terms associated with MetS research in digital health care; second, to establish a text network comprising core keywords from existing literature on MetS and digital health care; and third, to identify trends in MetS research within the digital health care context through topic modeling.

## Methods

### Study Design

This is a descriptive study in which we used topic modeling techniques to classify keywords into topics within the realm of digital health care for MetS management and tracked topic-specific trends over time.

### Search Strategy and Data Collection

We conducted a comprehensive search of 6 electronic academic databases, namely PubMed, Embase, Cochrane, CINAHL, SCOPUS, and Web of Science. The search strategy involved using combinations of various terms to filter publication titles, abstracts, and keywords. The terms used included: (Digital health OR mobile health OR ehealth OR e-health OR mhealth OR telehealth OR electronic health OR web-based OR apps OR wearables OR devices) AND (metabolic syndrome OR Metabolic Syndromes OR syndrome, metabolic OR syndromes, metabolic OR MetS OR syndrome X) (Multimedia Appendix 1).

The search and selection processes were performed in May 2023. We included all abstracts from articles published before April 20, 2023. There were no restrictions on the start date of the literature to ensure a comprehensive analysis of studies over time. Studies were excluded if they involved participants with conditions other than MetS or conditions related to subdiagnosis criteria such as obesity, diabetes, hypertension, and dyslipidemia. Additionally, studies without an abstract or with a non-English abstract were excluded.

**Figure 1:**
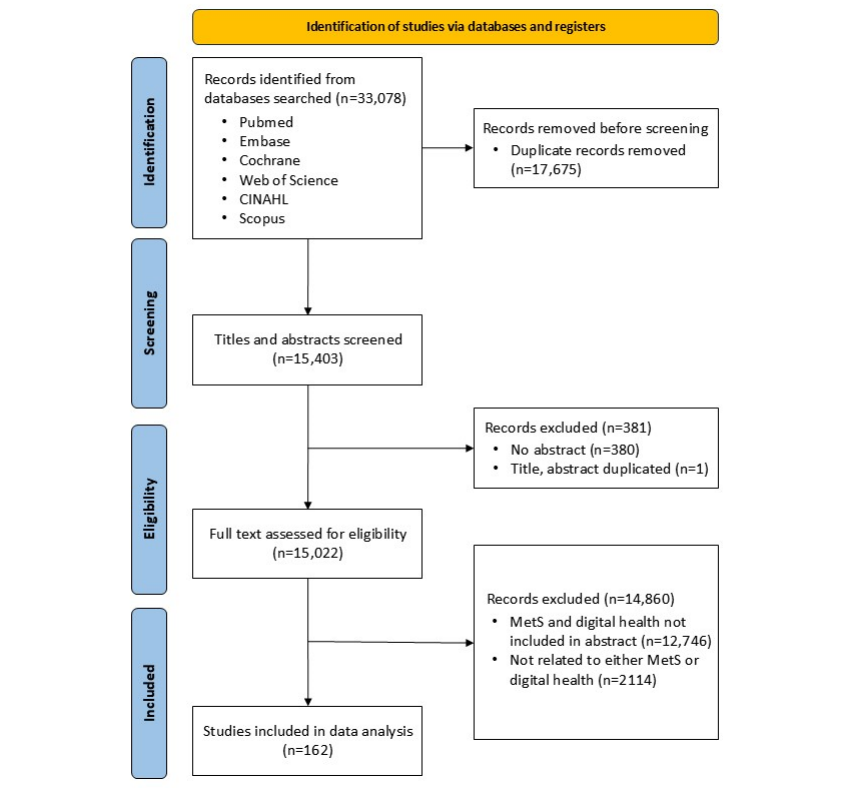
Flowchart of the study selection process.

From the initial 33,078 studies identified, 17,675 duplicate entries were removed. The remaining 15,403 studies were screened based on their title and abstract. Studies were further excluded based on the following criteria: 12,746 studies lacked keywords related to both MetS and digital health care, 2114 studies did not focus on patients, 380 studies lacked an abstract, and one was a duplicate. Subsequently, 2 researchers independently screened the identified articles’ titles and abstracts. In cases of disagreement, consensus was reached through discussion and further review against the selection criteria. Ultimately, a total of 162 articles were included for data analysis (Figure 1). Data, including serial numbers, authors, titles, author keywords, and abstracts, were organized into a matrix using Microsoft Excel.

### Analysis

We performed text preprocessing, topic modeling, and text network analysis on the collected abstracts. Custom code written in Python was used to execute these tasks. The code can be reviewed on GitHub [19].

#### Text Preprocessing

In the text preprocessing stage, we initiated the process by removing internet address formats, HTML tags, and special characters from the abstracts. Additionally, any text that was not in English was discarded. We then harmonized expressions that had similar or identical meanings; for instance, the terms “mobile health,” “mhealth,” and “m-health” were consolidated as “m-health.” We also compiled a list of specialized terms to avoid issues with word separation because of spacing; notably, the term “randomized controlled trial” was transformed into “randomized_controlled_trial.” To refine the focus of our analysis, we carefully curated a list of stop words by iteratively reviewing the text analysis results. This process allowed us to differentiate between common terms and significant terms, thus ensuring that essential information was retained. Consequently, we excluded typical English stop words as well as frequently occurring domain-specific terms such as “study,” “result,” and “research.” The aim was to prioritize in-depth analysis over a more generalized examination. To standardize different word forms like -ing, -ed, and -s, we used lemmatization. The preprocessing stage concluded with the adoption of noun extraction as a standard text mining practice to augment semantic understanding.

#### Text Network Analysis

In text network analysis, words act as nodes, and their connections form edges, thereby enabling semantic analysis. In this study, we established a “co-occurrence” relationship among nouns present in the abstracts of scholarly articles, representing these relationships as edges in the network. Focusing on the specific keyword “metabolic_syndrome,” we constructed a network based on the frequency of word co-occurrence, limiting edges to instances where words appeared together at least twice. We also allocated higher weights to edges where word co-occurrence was repeated.

Our network construction resulted in a total of 1047 nodes and 34,377 edges. Using this foundational data, we evaluated the degree, closeness, and betweenness centrality for each node. Degree centrality quantifies the number of edges linked to a given node, thus indicating its direct connections to other words within the network. Closeness centrality is a measure of how quickly a node can reach all other nodes in the network, reflecting its proximity to a multitude of other terms. Betweenness centrality measures the frequency with which a node resides on the shortest paths between other nodes in the network, highlighting its function as a connector or bridge among words.

Given the substantial number of nodes and edges, the network inherently possesses the characteristics of a dense graph, complicating the task of visualization. To address this issue, we chose to visualize only the top 5% of nodes based on their degree centrality, which yielded 52 nodes, and focused our analysis on keywords strongly associated with “metabolic syndrome.” For both network construction and visualization, we used the Python library network to analyze the degree centralization index.

#### Topic Modeling

Topic modeling is a text mining technique for elucidating the inherent semantic structure of textual data. We performed topic modeling on a refined set of abstracts to uncover the latent themes within them. We included only abstracts in our analysis because topic modeling has been shown to be particularly effective with short texts [20]. Classical algorithms for this purpose include latent semantic analysis and latent Dirichlet allocation. These traditional methods rely on statistics-based word embeddings, which often ignore the order and relationships between words within the text. More advanced models, such as the combined topic model [21] and BERTopic [22], have recently been introduced for this task. In this study, we used the BERTopic model, which uses BERT (Bidirectional Encoder Representations from Transformers), a large language model, to perform semantic-based embeddings. BERT captures the contextual relationships between words by considering their order and entire sentence context through mechanisms like attention. This allows BERTopic to extract topics that are sensitive to the relationships between words, leading to more nuanced and contextually aware topic modeling. Our implementation was conducted using the BERTopic API (application programming interface) [23].

The BERTopic API is designed for topic modeling, leveraging BERT embeddings and clustering algorithms to extract coherent topics from textual data. It uses large language models like BERT to capture the semantic relationships between words and then applies techniques such as hierarchical density–based clustering of applications with noise for clustering and term frequency-inverse document frequency for keyword extraction. This combination allows BERTopic to generate interpretable topics that reflect the underlying themes in the data, making it a powerful tool for analyzing and understanding large text corpora. We used the “all-mpnet-base-v2” large language model, which has been fine-tuned on over a billion sentences and was chosen for its top performance in sentence embeddings [24]. Using BERTopic, we conducted topic analysis on a dataset comprising 162 abstracts, which led to the extraction of 13 distinct topics. Among these, 1 topic contained outlier values that presented challenges for semantic interpretation. To address this, we amalgamated the documents associated with this outlier topic into the nearest topic cluster, resulting in a final count of 12 topics. Each topic was represented by a set of words, which we closely examined along with the papers related to each topic to assign them appropriate names.

## Results

### Core Keywords

**Figure 2:**
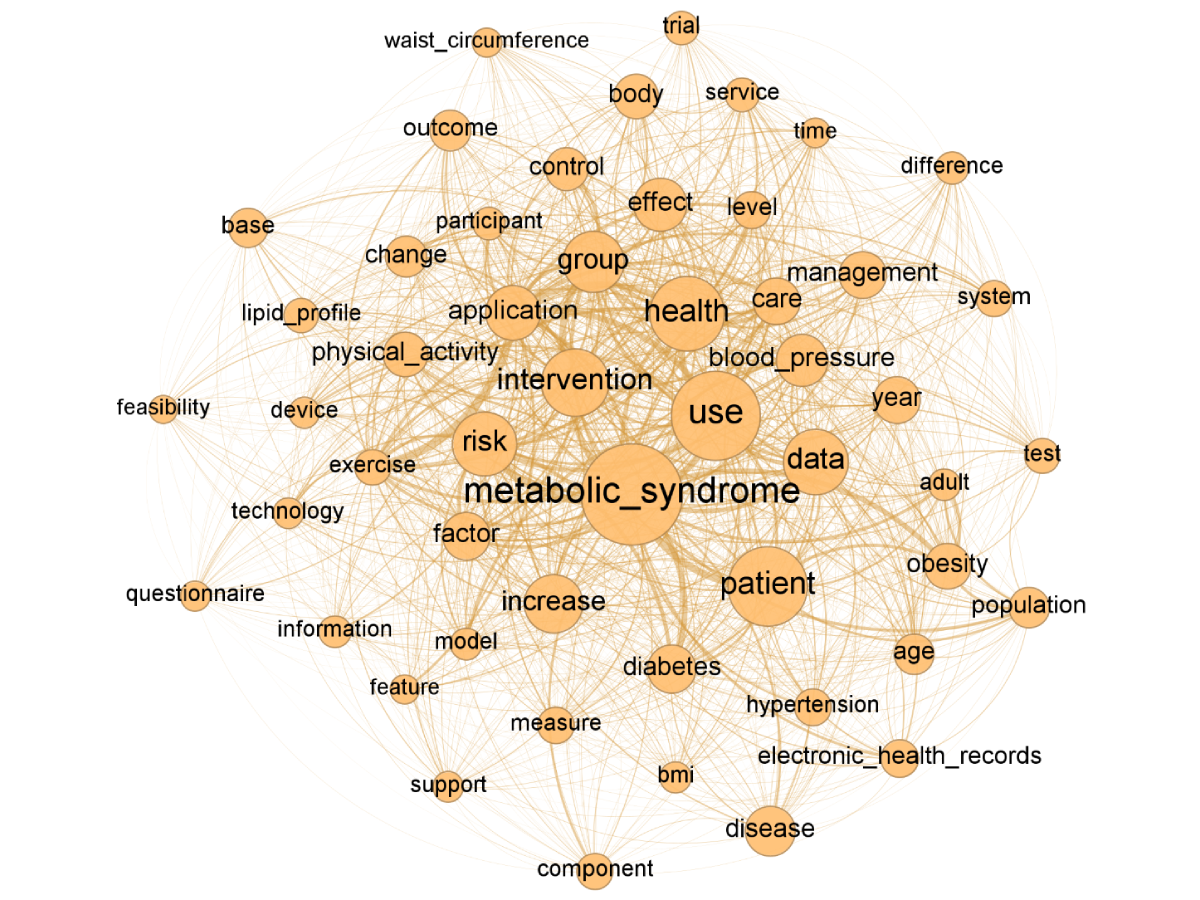
Network of top 5% keywords based on degree centrality.

A network comprising 1047 nodes (keywords) and 34,377 edges was constructed from the 162 abstracts. Table 1 presents the core keywords associated with MetS in the context of digital health care. The top 10 keywords, ranked by edge count, are as follows: “MetS” (817 edges), “use” (n=724), “patient” (n=644), “health” (n=610), “intervention” (n=555), “data” (n=536), “risk” (n=526), “group” (n=503), “increase” (n=483), and “application” (n=456). Figure 2 presents the 817 keywords that co-occur with “MetS,” which has the highest degree of centrality. The degree centrality of the keywords, in descending order, are: “MetS” (0.781), “use” (0.692), “patient” (0.616), “health” (0.583), “intervention” (0.531), “data” (0.512), “risk” (0.503), “group” (0.481), “increase” (0.463), and “application” (0.436). The closeness centrality measures for the keywords are: “MetS” (0.819), “use” (0.764), “patient” (0.721), “health” (0.705), “intervention” (0.680), “data” (0.671), “risk” (0.667), “group” (0.657), “increase” (0.650), and “application” (0.639). The betweenness centrality of the keywords, in descending order, are “MetS” (0.117), “use” (0.071), “patient” (0.066), “health” (0.042), “intervention” (0.034), “data” (0.034), “risk” (0.030), “group” (0.029), “increase” (0.024), and “application” (0.020).

**Table 1:** Core keywords in MetS research within digital health care.

Rank	Keywords	Degree	Degree centrality	Closeness centrality	Betweenness centrality
1	MetS	817	0.781	0.819	0.117
2	use	724	0.692	0.764	0.071
3	patient	644	0.616	0.721	0.066
4	health	610	0.583	0.705	0.042
5	intervention	555	0.531	0.680	0.034
6	data	536	0.512	0.671	0.034
7	risk	526	0.503	0.667	0.030
8	group	503	0.481	0.657	0.029
9	increase	484	0.463	0.650	0.024
10	application	456	0.436	0.639	0.020
11	effect	444	0.424	0.634	0.018
12	blood pressure	432	0.413	0.629	0.017
13	disease	416	0.398	0.623	0.016
14	diabetes	403	0.385	0.619	0.015
15	factor	399	0.381	0.617	0.014
16	care	395	0.378	0.616	0.013
17	management	393	0.376	0.615	0.012
18	year	391	0.374	0.614	0.012
19	obesity	384	0.367	0.612	0.011
20	body	376	0.359	0.609	0.011
21	physical activity	376	0.359	0.609	0.010
22	control	360	0.344	0.603	0.010
23	change	348	0.333	0.599	0.009
24	outcome	343	0.328	0.597	0.009
25	population	341	0.326	0.597	0.009

### Text Network Analysis

**Figure 3:**
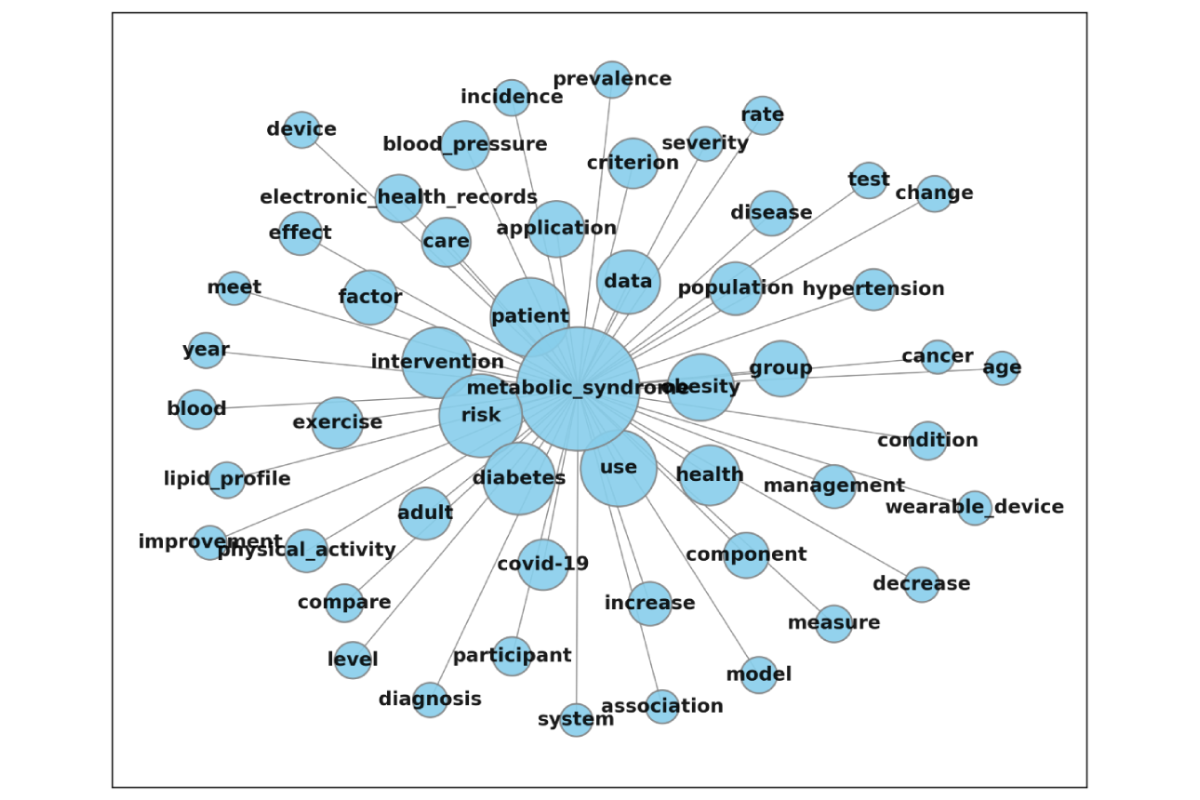
Network of core keywords related to MetS. MetS: metabolic syndrome.

Using the core keywords extracted from the abstracts, a text network analysis was conducted to visualize relationships among these core keywords. To enhance understanding of the keyword structure, we visualized the network of top keywords based on degree centrality, highlighting the top 5% of nodes (Figure 2). “MetS,” the keyword most frequently connected to others, occupied the central position within the network. Moreover, a network of core keywords was constructed by organizing keywords in proximity to “MetS” based on their frequency of co-occurrence with it (Figure 3). The top 5 keywords frequently co-occurring with “MetS” were “risk,” “patient,” “diabetes,” “intervention,” and “use.”

### Topic Modeling

Table 2 presents the distribution of keywords related to MetS in digital health care studies, organized by topic. Each topic was identified and labeled based on its associated keywords. Topic 1 constituted 19.8% of all articles and featured the top 10 keywords, in descending order of probability, as follows: “telehealth,” “diabetes,” “control,” “intervention,” “self-management,” “care,” “effect,” “group,” “type,” and “health.” Topic 1 was labeled “use of telehealth for self-management intervention of diabetes control.” Topic 2 comprised 14.2% of the total articles, with its top 10 keywords being “MetS,” “electronic health records,” “diabetes,” “patients,” “complication,” “risk,” “criterion,” “data,” “population,” and “model.” Topic 2 was labeled “use of EHR to manage MetS complications.” Topic 3, which accounted for 12.3% of all articles, was named “use of wearable devices for monitoring metabolic status.” The top 10 keywords for this topic included “device,” “wearable device,” “use,” “sensor,” “technology,” “monitoring,” “data,” “sit,” “disease,” and “review.” Topic 4 represented 10.5% of the articles and was labeled “use of apps for weight loss intervention.” Its top 10 keywords were “obesity,” “weight loss,” “intervention,” “program,” “health,” “body,” “application,” “weight,” “effect,” and “group.” Topic 5, making up 6.8% of the articles, was named “use of digital health for health-related behavior-changing intervention.” The top 10 keywords for this topic were “intervention,” “application,” “participant,” “hospitalization,” “care,” “change,” “m-health,” “trial,” “effect,” and “behavior.” Topic 6, accounting for 6.2% of all articles, was labeled “use of digital health to assess MetS status and trend during COVID-19.” Its top 10 keywords were “COVID-19,” “diabetes,” “MetS,” “patient,” “data,” “disease,” “health,” “disorder,” “incidence,” and “decrease.” Topic 7, constituting 7.4% of the articles, was named “use of digital health for blood pressure control with physical activity.” The top 10 keywords were “blood pressure,” “factor,” “group,” “physical activity,” “risk,” “MetS,” “child,” “measure,” “intervention,” and “adult.” Topic 8, also comprising 7.4% of the articles, was labeled “use of apps for engaging in physical activity.” The top 10 keywords for this topic were “physical activity,” “application,” “intervention,” “minute,” “body,” “engagement,” “week,” “user,” “effect,” and “woman.” Topic 9, accounting for 6.2% of the articles, was named “use of apps for access to data on individual metabolic status.” The top 10 keywords were “application,” “user,” “health,” “use,” “access,” “care,” “management,” “data,” “information,” and “feature.” Topic 10, representing 4.3% of all articles, was labeled “use of m-health for diabetes control.” Its top 10 keywords were “application,” “diabetes,” “smartphone,” “field,” “modification,” “HbA_1c_,” “review,” “use,” “control,” and “search.” Topics 11 and 12 each accounted for 2.5% of the articles. Topic 11 was named “use of digital health for management of metabolic status in women of child-bearing age,” and its top 10 keywords were “woman,” “pregnancy,” “feedback,” “health,” “pilot,” “intervention,” “program,” “delivery,” “interview,” and “factor.” Topic 12, labeled “use of digital health for management of lipid profile,” featured the top 10 keywords “lipid profile,” “patient,” “training,” “optimization,” “level,” “therapy,” “health care,” “coach,” “create,” and “metabolism.”

**Table 2:** Grouping of topics and high-ranking keywords.

Key words	Topic 1	Topic 2	Topic 3	Topic 4	Topic 5	Topic 6	Topic 7	Topic 8	Topic 9	Topic 10	Topic 11	Topic 12
1	Telehealth	MetS^a^	Device	Obesity	Intervention	COVID-19	Blood pressure	Physical activity	Application	Application	Woman	Lipid profile
2	Diabetes	EHR^b^	Wearable device	Weight loss	Application	Diabetes	Factor	Application	User	Diabetes	Pregnancy	Patent
3	Control	Diabetes	Use	Intervention	Participant	MetS	Group	Intervention	Health	Smartphone	Feedback	Training
4	Intervention	Patients	Sensor	Program	Hospitalization	Patient	Physical activity	Minute	Use	Field	Health	Optimization
5	Self-management	Complication	Technology	Health	Care	Data	Risk	Body	Access	Modification	Pilot	Level
6	Care	Risk	Monitoring	Body	Change	Disease	MetS	Engagement	Care	HbA_1c_	Intervention	Therapy
7	Effect	Criterion	Data	Application	mHealth^c^	Health	Child	Week	Management	Review	Program	Health care
8	Group	Data	Sit	Weight	Trial	Disorder	Measure	User	Data	Use	Delivery	Coach
9	Type	Population	Disease	Effect	Effect	Incidence	Intervention	Effect	Information	Control	Interview	Create
10	Health	Model	Review	Behavior	Behavior	Decrease	Adult	Woman	Feature	Search	Factor	Metabolism

^a^MetS: metabolic syndrome.

^b^EHR: electronic health records.

^c^mHealth: mobile health.

### Topic Trend

**Figure 4:**
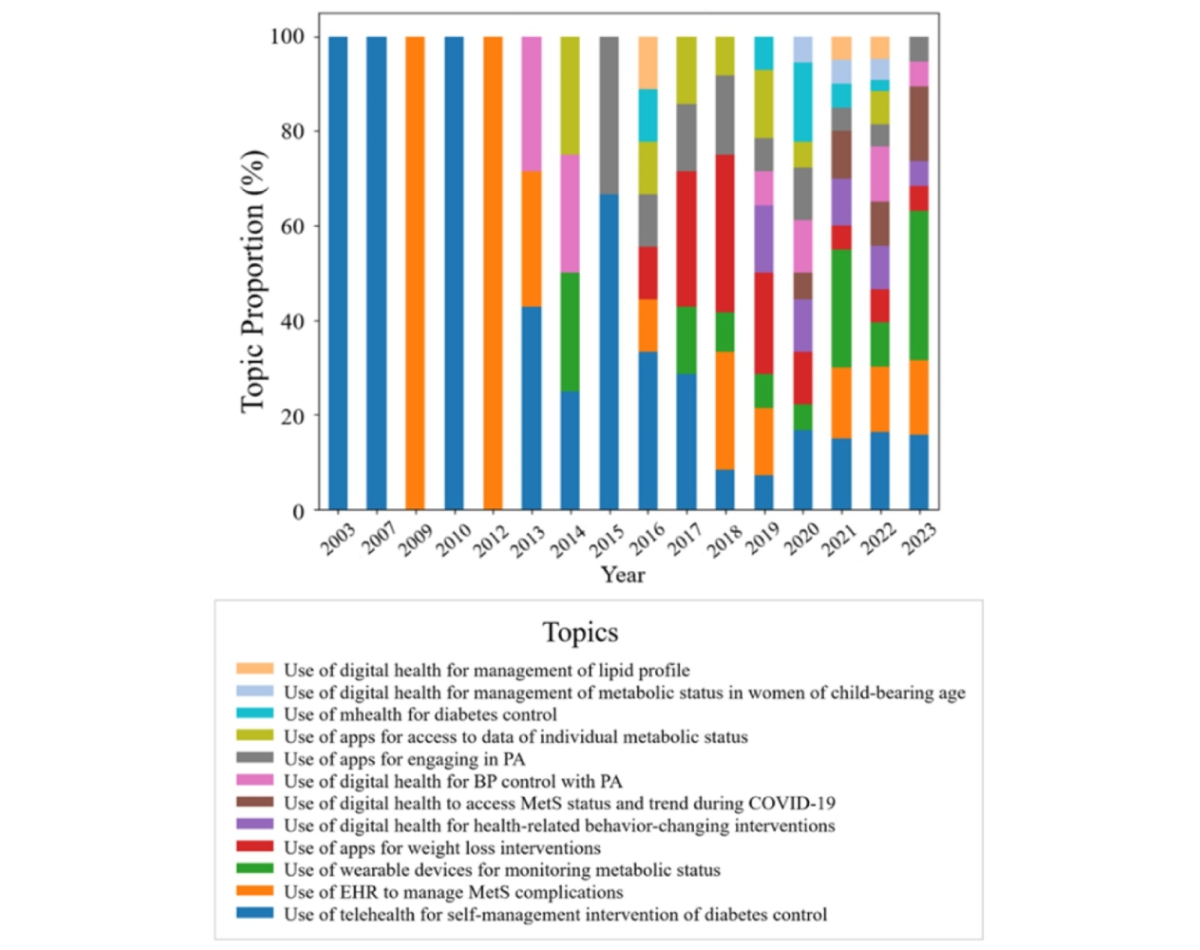
Annual trend in topics. BP: blood pressure; EHR: electronic health record; MetS: metabolic syndrome; PA: physical activity.

Research into MetS within the context of digital health care began in 2003 and showed steady growth over time, with a substantial surge in publications observed after the onset of COVID-19. This trend was particularly notable between 2021 and 2022, when the number of related articles rose sharply from 20 to 43, reflecting heightened interest and activity in this field. In the early years, the focus of research was primarily on telemedicine and electronic medical records. However, the scope of studies has since broadened to include a wider array of topics, such as the development of intervention programs that leverage wearable devices and mobile apps to manage subjects’ metabolic status and promote their engagement in health-related behaviors. Moreover, beginning in 2020, research has increasingly targeted specific populations, including women of childbearing age. The emergence of COVID-19 also prompted investigations into the application of digital health care in remote scenarios necessitated by the pandemic (Figure 4).

## Discussion

### Principal Results

Regarding key terms in the digital health care domain related to MetS, the following terms emerged as predominant: “MetS,” “use,” “patient,” “health,” “intervention,” “data,” “risk,” “group,” “increase,” “application,” and “effect.” Given that MetS was the focal point of our inquiry, it unsurprisingly ranked highest in both degree and centrality among the keywords. Our analysis further suggests that the primary research focus in this area involves the collection of data to verify the effectiveness of digital health interventions, particularly those delivered via apps targeting specific patient groups for MetS management. This observation was corroborated by text network analysis (Figures 2 and 3).

Additionally, an analysis of the number of relevant studies published annually revealed a noticeable increase in publications, particularly following the onset of the COVID-19 pandemic. While digital health care has been used for MetS management for several years, the COVID-19 crisis marked a significant turning point, enhancing both its accessibility and applicability. The term “COVID-19” itself became a key focus in topic 6 (Use of digital health to assess MetS status and trends during the COVID-19 pandemic), first emerging in the literature in 2020. Given these developments, we can reasonably anticipate an escalation in research activity surrounding MetS and digital health care applications in the coming years. This is particularly pertinent as the prevalence of MetS continues to rise, necessitating lifestyle alterations to mitigate or reverse such trends [1,25]. Moreover, as the digital health care field expands in tandem with technological advancements [7,26], we expect this upward trajectory to continue.

Furthermore, we observed a significant disparity in the representation of individual topics. Topic 1, which focuses on the use of telehealth for self-management in diabetes control, constitutes 19.8% of the studies. In contrast, topic 11, which explores the use of digital health for managing metabolic status in women of childbearing age, and topic 12, which centers on the use of digital health for lipid profile management, each make up only 2.5%. This distribution suggests that not all topics are equally represented in the literature. However, it is worth noting that topic 11 was first introduced in 2020 and topic 12 in 2016, making them relatively new areas of research. This may account for their lower representation. In comparison, topic 1 has been under consistent since 2003, indicating that the self-management of diabetes remains a long-standing primary concern in this field.

Some topics and their associated keywords align with the burgeoning field of precision medicine. Precision medicine aims to provide personalized care to each individual by focusing on the individual’s genomic structure and molecular biology. In this context, health care providers make medical decisions based on the unique characteristics of each individual rather than generalizing from average patients [27,28]. Given this focus, there is a growing need for intervention studies that target specific subjects, a need that is mirrored by the keywords identified in this study’s results. For example, topic 9 (Use of apps for access to data of individual metabolic status) centers on individuals’ metabolic status and emerged in the literature in 2014, with research ongoing through 2022. Keywords associated with topic 7 (Use of digital health for BP control with PA) include “child” and “adult,” while those for topic 8 (Use of apps for engaging in PA) feature the term “woman.” Topic 11 (Use of digital health for management of metabolic status in women of childbearing age) includes keywords like “woman,” “pregnancy,” and “delivery,” reflecting its focus on women who are pregnant or in the process of childbirth. This targeted research contributes incrementally to the developing knowledge base for tailored interventions, which aligns with the broader medical community’s emphasis on precision medicine. Nonetheless, given the small proportion of these topics, further research is essential because precision medicine relies on comprehensive population-level data to inform the selection of the most appropriate treatment for a given individual [27].

### Limitations

This study presents some limitations that warrant attention. First, the dataset comprises a relatively small number of abstracts, which may impact the generalizability of the findings. To collect articles for use in our analysis, the topic search and category settings were retrieved on academic databases. It is possible that the data collected did not include all papers related to MetS in digital health care. Second, for the sake of clarity in interpretation, the analysis focused solely on nouns. The exclusion of other word types such as adjectives and adverbs may result in differing outcomes compared with analyses that consider all word types.

### Conclusions

This study used topic modeling, a data-mining method, to extract salient topics related to MetS and digital health care. By analyzing historical research trends, the study aims to forecast and establish directions for future research endeavors. To this end, we systematically gathered all pertinent data from several academic databases, conducted data cleaning, implemented topic modeling, and undertook visualization and analysis of the results. This research contributes to a more comprehensive understanding of MetS management within the context of digital health care and offers valuable insights for future researchers in this domain.

## Appendix

Multimedia Appendix 1 Search Strategy.

## Abbreviations

API: application programming interface
BERT: Bidirectional Encoder Representations from Transformers
MetS: metabolic syndrome
PA: physical activity

## Data Availability

The datasets generated or analyzed during this study are available from the corresponding author on reasonable request.
