# Supplementary material for: Research Trends on Metabolic Syndrome in Digital Health Care Using Topic Modeling: Systematic Search of Abstracts
Source: J Med Internet Res. 2024 Dec 12;26:e53873. doi: 10.2196/53873 (PMC11671787; doi:10.2196/53873)
Supplement: Multimedia Appendix 1 [file jmir_v26i1e53873_app1.docx]

**Multimedia Appendix 1.** Search Strategy

| **Database** | **Search No.** | **Query** |
| --- | --- | --- |
| **PubMed (MeSH)** | #1 | “digital health”[tiab] OR “mobile health”[tiab] OR “ehealth”[tiab] OR “e-health”[tiab] OR “mhealth”[tiab] OR “telehealth”[tiab] OR “electronic health”[tiab] OR “web based”[tiab] OR “apps”[tiab] OR “wearables”[tiab] OR “devices”[tiab] |
|  | #2 | “metabolic syndrome”[Majr] OR “Metabolic Syndromes”[Majr] OR “syndrome, metabolic”[Majr] OR “syndromes, metabolic”[Majr] OR “MetS”[Majr] OR “syndrome X”[Majr] |
|  | #3 | (intervention[tiab] OR “Program” [Publication Type] OR program[tiab]) |
|  | #4 | #1 AND #2 AND #3 |
| **Embase (Emtree)** | #1 | digital health’ ,ab OR ‘mobile health’ ,ab OR ‘ehealth’ ,ab OR ‘e-health’ ,ab OR ‘mhealth’ ,ab OR ‘telehealth’ ,ab OR ‘electronic health’ ,ab OR ‘web based’ ,ab OR ‘apps’ ,ab OR ‘wearables’ ,ab OR ‘devices’ ,ab |
|  | #2 | metabolic syndrome’/mj OR ‘metabolic syndromes’ ,ab OR ‘syndrome, metabolic’ ,ab OR ‘syndromes, metabolic’ ,ab OR ‘MetS’ ,ab OR ‘syndrome X’ ,ab |
|  | #3 | #1 AND #2 |
| **Cochrane (MeSH)** | #1 | MeSH descriptor: [digital health] explode all trees |
|  | #2 | (“digital health” OR “mobile health” OR “ehealth” OR “e-health” OR “mhealth” OR “telehealth” OR “electronic health” OR “web based” OR “apps” OR “wearables” OR “devices”) ,ab |
|  | #3 | #1 OR #2 |
|  | #4 | MeSH descriptor: [Metabolic Syndrome] explode all trees |
|  | #5 | (“metabolic syndrome” OR “Metabolic Syndromes” OR “syndrome, metabolic” OR “syndromes, metabolic” OR “MetS” OR “syndrome X”) ,ab |
|  | #6 | #4 OR #5 |
|  | #7 | #3 AND #6 |
| **CINAHL (CINAHL Headings)** | #1 | (MM “digital health”) |
|  | #2 | TI (“digital health” OR “mobile health” OR “ehealth” OR “e-health” OR “mhealth” OR “telehealth” OR “electronic health” OR “web based” OR “apps” OR “wearables” OR “devices”) |
|  | #3 | #1 OR #2 |
|  | **#4** | (MM “Metabolic Syndrome+”) |
|  | #5 | TI (“metabolic syndrome” OR “Metabolic Syndromes” OR “syndrome, metabolic” OR “syndromes, metabolic” OR “MetS” OR “syndrome X”) |
|  | #6 | S4 OR S5 |
|  | #7 | S3 AND S6 |
| **SCOPUS** | #1 | (“digital health” OR “mobile health” OR “ehealth” OR “e-health” OR “mhealth” OR “telehealth” OR “electronic health” OR “web based” OR “apps” OR “wearables” OR “devices”) AND (“metabolic syndrome” OR “Metabolic Syndromes” OR “syndrome, metabolic” OR “syndromes, metabolic” OR “MetS” OR “syndrome X”) |
| **Web of Science** | #1 | (“digital health” OR “mobile health” OR “ehealth” OR “e-health” OR “mhealth” OR “telehealth” OR “electronic health” OR “web based” OR “apps” OR “wearables” OR “devices”) AND (“metabolic syndrome” OR “Metabolic Syndromes” OR “syndrome, metabolic” OR “syndromes, metabolic” OR “MetS” OR “syndrome X”) |
